# Updating the Surgical Preference List

**DOI:** 10.7759/cureus.2997

**Published:** 2018-07-18

**Authors:** James S Huntley, Jason J Howard, Jason Simpson, David L Sigalet

**Affiliations:** 1 Department of Surgery, Sidra Medicine, Ar-Rayyan, QAT; 2 Department of Perioperative Care, Sidra Medicine, Ar-Rayyan, QAT

**Keywords:** preference list, pick list, fad list, standardization, surgery

## Abstract

Surgical procedure ‘preference lists’ are used worldwide, but their practice varies widely. Despite being positioned at a critical point in a surgical care pathway, they are often underemphasized, poorly maintained, and substandard. The following editorial material is gleaned from our experience in the set-up of a tertiary hospital on a green field site in Qatar. We comment on the use of preference lists, and contend that focus on standardizing and maintaining preference lists within an electronic record affords substantial opportunities for cost containment, whilst adding efficiency, safety, and value. We believe this approach represents an ‘easy win’ which would be applicable elsewhere.

## Editorial

Surgical procedure preference lists (pick- or Fad-lists) are a valuable feature of operating practice, and they have the potential to be ‘the nexus of an efficient OR’ [1]. However, presumably reflecting local practices, they have evolved somewhat divergently in different countries and institutions. Worldwide, many hospitals still have hardcopy pick-list folders individualized by surgeons, and these are frequently substandard, poorly maintained/incomplete, out of date, and replete with ambiguous or incorrect details [2]. Though they are generated with the best of intentions and are generally helpful, they may nevertheless themselves be a source of error [2].

At several institutions, surgical practice has undergone increased standardization, partly by focusing on departmental, as opposed to individual, preference lists [2-3], e.g., for appendectomy [4] and laparoscopic adult cholecystectomy [5]. This strategy allows rationalizing equipment and procedure, minimizing costs, and increasing safety and value. Standardization of pick-lists and a dialogue between users about cost control can achieve substantial savings for disposable supplies, as well as streamline for efficiency and maintenance [5]. This standardization has the potential to decrease the amount of consumable wastage by opening only what is necessary and agreed upon by the end-user as a standard. In addition, standardization of instrument trays by preference list decreases costs related to sterilization, and reduces the risk of nonavailability of trays. Cost containment is increasingly important in an era of fiscal responsibility. Even when not standardized, preference list maintenance produces savings.

We use a pragmatic comprehensive template (Table 1) for the preference list, incorporating a 'Special notes/Surgical preferences' section, so that individualization of procedures is easily accomodated (any quirks/positive deviations of a particular surgeon's practice can be highlighted) within the standard protocol. This removes the need for the maintenance of a large number of cards (each procedure for each surgeon). Additionally, preference lists sit within the electronic record, allowing for updation (modification/improvement) after protocolized review at the sign-out of every case; changes can be made by a technical operator within 24 hours.

**Table 1:** Standardized preference card template (left column in bold) – with corresponding example of tarsal coalition resection.

Division	Orthopedics
Definition/code	OPC.6: A.25
Procedure (narrative)	Tarsal coalition resection - calcaneonavicular
Patient preparation	Nil
Medication	Preoperative antibiotic: cefotaxime
Diagnostic imaging	2D C-arm + C-Armour drape
Lab requirements - preoperative	-
Lab requirements - operative	-
Room/table set-up	Radiolucent table
Patient position	Supine; feet at end of table; gel bolster under ipsilateral buttock as appropriate
Skin preparation	Chlorhexidine
Draping	Split drape + ¾-drape
Cautery settings	Diathermy - unipolar, spatula-tip – settings at surgeon’s advice
Equipment/instrumentation	Upper thigh tourniquet; Ortho pan 1; Laminar spreaders; Osteotomes and gauges; Sagittal saw; Pituitary rongeurs
Sterile supplies	Nondisposable light handles; smoke evacuator; small suction tube
Closure	4/0 Monocryl
Dressings/cast	Inadine + blue gauze/BK POP cast
Special notes/surgical preferences	JSH/JJH - bone wax; narrow Hohman retractors

At a pragmatic level, Dizon et al. [1] expose some of the vulnerabilities of using the preference list as a central point in the electronic record and ordering process, again emphasizing on list maintenance and completeness. Proper co-ordination with the automated carousel system (or similar system) for instrument and consumable delivery is also important [1]. These authors note that a computer-based preference list system has several potential attributes: (i) efficient resource allocation, (ii) efficient preparation of the operation room, (iii) effective cost control, (iv) decreased delay to surgeries, (v) better inventory control and efficiency, and (vi) reliable charging. We would add the benefits potential for standardization (reducing variability according to individual surgeon), and flexibility (ease of modification/alterations, as long as a system for this is in place from the outset).

Although uploading and maintaining the data is a substantial initial investment and ongoing commitment, the preference list has the potential to sit at a pivotal site in the overall surgical care pathway, itself vaunted to minimize unwanted variation in a complex environment. We believe our preference card system to be widely applicable, and an easy ‘win’: (i) optimizing the economics of inventory and practice, (ii) synergistic with the WHO checklist, and (iii) maximizing safety, theatre efficiency, and value.
